# An epidemiological study of suspected rabies exposures and adherence to rabies post-exposure prophylaxis in Eastern Thailand, 2015

**DOI:** 10.1371/journal.pntd.0007248

**Published:** 2020-02-27

**Authors:** Onphirul Yurachai, Soawapak Hinjoy, Ryan M. Wallace

**Affiliations:** 1 Field Epidemiology Training Program (FETP), Bureau of Epidemiology, Department of Disease Control, Ministry of Public Health, Thailand; 2 Active Surveillance Section, Bureau of Epidemiology, Department of Disease Control, Ministry of Public Health, Thailand; 3 Poxvirus and Rabies Branch, Centers for Disease Control and Prevention, Atlanta, Georgia, United States of America; Universidad Nacional Mayor de San Marcos, PERU

## Abstract

**Background:**

Human rabies is a notifiable condition in Thailand, and 46 confirmed and probable cases were reported from 2010–2015; eleven were reported from Eastern Thailand. Although rabies is vaccine preventable, more than 90% of persons who died of rabies in Thailand either did not receive or inappropriately discontinued post-exposure prophylaxis (PEP). In 2012 Thailand launched a national animal rabies elimination program with the goal of elimination by 2020. One of the policies of this national program is to improve detection of animal rabies exposures, access to PEP, and adherence to vaccine schedules. To achieve this goal, several hospital-based electronic PEP surveillance systems have been instituted throughout Thailand.

**Method:**

Data from a voluntary, electronic hospital-based, rabies exposure and PEP surveillance system was analyzed from eight provinces in Eastern Thailand for the time period January 1 –December 31, 2015. The surveillance system collects data from all persons who present to an R36-integrated healthcare facility with a suspected rabies exposure, including characteristics of the biting animals, categorization of the rabies exposure, and adherence to PEP recommendations. The crude rate of healthcare seeking for a suspected rabies exposure was assessed by province, and a multivariable linear regression model was developed to determine the potential extent of undetected rabies exposures due to bite treatment at healthcare facilities that do not utilize the R36 system. Suspected rabies exposures were described by patient demographics, location of wound, and disposition of the offending animal. A comparison of adherence to intramuscular and intradermal vaccination regimens was performed and odds ratios were calculated for factors related to unadvised PEP discontinuation.

**Result:**

6,204 suspected rabies exposures were reported from eight Eastern Thailand provinces, yielding a crude exposure rate of 106 reported rabies exposures per 100,000 population. When adjusted for under-detection due to non-participating hospitals and province-level demographic differences, the estimated suspected rabies exposure rate was 204/100,000. Dogs were the main source of exposure (77.8%) and children age <15 years and elderly age >60 years had the highest overall reported exposure rate (189.7 and 189.2/100,000). Adherence to either the intramuscular 5-dose or the intradermal 4-dose PEP regimen was low (15.8% and 46.5%, respectively); rabies immunoglobulin was received by only 15% of persons for whom it was indicated. Persons with rabies exposures were more likely to discontinue the vaccination series against medical advice if they were male, aged 16–45, if they received immunoglobulin, or if received the intramuscular regimen.

**Conclusion:**

When adjusting for number of reporting hospitals, province population density, number of hospitals per population and average family income, the expected report rate increased 1.9-fold, indicating that there is likely a high level of under-detection of persons seeking medical care for suspected rabies exposures. Expanded implementation of electronic surveillance systems will likely improve reporting and the epidemiologic knowledge of rabies exposures. Analysis of data collected from this system revealed very low rates of adherence to rabies vaccination recommendations. PEP adherence was better by the intradermal route, which provides more support for its use in situations where it is economically feasible.

## Author summary

While rabies is a vaccine preventable disease, and despite tremendous improvements in access to healthcare, human rabies fatalities continue to occur in Thailand. Data from a voluntary, electronic hospital-based, rabies exposure and PEP surveillance system was analyzed from eight provinces in Eastern Thailand. In total 6,204 suspected rabies exposures were reported between January 1 –December 31, 2015, yielding a crude exposure rate of 106 reported rabies exposures per 100,000 population or 204/100,000 when adjusted for under-reporting. Dogs were the main sources of exposure and children age < 15 years and elderly aged > 60 years had the highest overall reported exposure rate. Adherence to either the intramuscular or the intradermal PEP regimen was low (15.8% for IM and 46.5% for ID). There is likely a high level of under-detection of persons seeking medical care for suspected rabies exposures in Eastern Thailand, but expanded implementation of electronic surveillance systems will likely improve reporting. Further investigation is needed to understand why rates of adherence to PEP were low. Adherence was better by the intradermal route, which provides more support for its use in situations where it is economically feasible.

## Introduction

Rabies virus causes a fatal encephalitis in mammals. Deaths from rabies can be prevented if post-exposure prophylaxis (PEP) is initiated soon after exposure and prior to symptom onset [1]. Rabies virus is most often transmitted through a bite or contact with saliva from a rabid animal. The World Health organization (WHO) estimates rabies virus causes 59,000 deaths annually with approximately 3.7 million DALYs [2]. Timely access to rabies PEP after an exposure is critical to prevent these human deaths [3–4] [5]. Barriers to timely vaccination, such as poverty and distance to medical facilities, can lengthen delays to initiating PEP and increase risk of death [3]. Rabies has been well-managed in Thailand, with only 46 human rabies deaths between 2010–2015 [6,7]. This has largely been achieved through improved access to rabies PEP; currently Thailand provides more than 600,000 PEP treatments annually [8].

Human rabies is a notifiable event in Thailand, however, suspected rabies exposures are not. To improve the understanding of rabies exposures, three hospital-based electronic systems were developed, each of which captures related, but different factors. ICD10 is the most commonly used system in Thailand for capturing suspected rabies exposures, but it does not collect characteristics of the exposure or PEP decisions. An Injury Surveillance (IS) system collects more characteristics than ICD10, but is only implemented in 33 hospitals, nationwide. The web-based R36 system was established in 2004 and collects detailed information for all individuals presenting for a suspected rabies exposure, as well as the PEP recommendations and adherence to those recommendations. R36 is a voluntary, hospital-based reporting platform that is used by 820 hospitals throughout Thailand [9].

Thailand utilizes the World Health Organization (WHO) rabies exposure categorization criteria as well as WHO PEP recommendations, although the latter have been slightly modified as shown in Table 1 [10–11]. Persons with category I exposures are not recommended PEP, and only recommended to wash the exposure site with soap and water. Persons with category II exposures are recommended to receive immediate vaccination, but are not advised to receive rabies immunoglobulin (RIG). Persons with category III exposures are recommended to receive vaccine and RIG as soon as possible. The regimen of PEP depends on vaccine type and route of administration (Table 1). Intramuscular (IM) vaccinations are given in 1 ml doses for HDCV, PCECV, PDEV or 0.5 ml doses for PVRV and CPRV; five doses over 30 days are recommended (1-1-1-1-1). Intradermal (ID) vaccinations are given in 0.1 ml doses at 2-sites, four times over 30 days (2-2-2-0-2). For previously complete pre-exposure vaccination or PEP, two doses of ID or IM are given three days apart (1-1-0-0-0). RIG is not necessary in such cases.

**Table 1:** Thailand rabies post exposure prophylaxis guideline [12].

Category	Rabies vaccination history and dosage					
	Never received (or received incomplete) PEP		Previously received complete pre-exposure vaccination or PEP			
			> 6 months since last PEP		< 6 months since last PEP	
	IM route	ID route	IM route	ID route	IM route	ID route
I	No treatment required. Only wash site of exposure with water and soap					
II	- Wound dressing - Vaccine on day 0, 3, 7, 14 and 30 - No RIG required	- Wound dressing - 0.1 ml. of vaccine 2-site on days 0, 3, 7 and 30 - No RIG required	- Wound dressing - Vaccine on day 0 and 3 - No RIG required	- Wound dressing - 0.1 ml. of vaccine 1-site on day 0 and 3 - No RIG required	- Wound dressing - Single shot of vaccine - No RIG required	- Wound dressing - 0.1 ml. single shot of vaccine 1-site - No RIG required
III	- Wound dressing - Vaccine on day 0, 3, 7, 14 and 30 - RIG required	- Wound dressing - 0.1 ml. of vaccine 2-site on day 0, 3, 7 and 30 - RIG required	- Wound dressing - Vaccine on day 0 and 3 - No RIG required	- Wound dressing - o.1 ml. of vaccine 1-site on day 0 and 3 - No RIG required	- Wound dressing - Single shot of vaccine - No RIG required	- Wound dressing - 0.1 ml. single shot of vaccine 1-site - No RIG required

**Note**: Dose of IM route 1 ml of HDCV, PCECV, PDEV or 0.5 ml of PVRV,CPRV

RIG = Immunoglobulin

RIG dosage: Equine RIG → 40 IU/ KG, Human RIG → 20 IU/KG

RIG should not be given if seven days have passed since initiating PEP

Immunocompromised patients or chloroquine drug receivers are not eligible for the ID route

If dog or cat is still alive 10 days after the exposure then the PEP can be discontinued

If the animal cannot be evaluated, full course of PEP should be administered

Thailand clinical practice guideline indicate that all victims who never received or received incomplete PEP should receive 5 doses of IM route

Three major systems have been used for providing healthcare in Thailand: 1) Universal health coverage; all people of Thailand must have a health identification card, called a “golden card”, which entitles them to any medical service for less than 30 baht per visit.[13] 2) Social Security Scheme; is financed by tripartite contributions from government, employers and employees. This scheme covers formal sector private employees for non-work related sickness, maternity and invalidity including cash benefits and funeral grants.[14] Around 20% (about 13 million) of the population has social security coverage including dependents of the Civil Servant, State Enterprise and Private School schemes [15] 3) Civil Servant Medical Benefit Scheme (CSMBS) and state enterprise benefit; are fully paid by the government and state enterprise. The Ministry of Finance is responsible for the CSMBS and the state enterprises are responsible for medical bills of their employees [16]. Rabies vaccination is free for the first visit, however the patient is responsible for the costs of vaccination for the remaining visits. Under certain situations these subsequent medical visits maybe be subsidized; persons with a government-provided golden card pay only 30 Thai bath per visit, and persons who are covered under the social security system are reimbursed for all costs.

In the past four years, the number of human rabies deaths in Thailand has dropped to less than 10 cases annually[17]. Over 90% of current human rabies deaths result from PEP non-compliance (did not initiate or discontinued PEP against medical advice) [13]. The primary function of R36 is to improve patient adherence to the PEP regimen through real-time electronic tracking of medical provider recommendations and hospital visits for vaccination. However, this system has never been used to analyze frequency or risk factors for rabies exposures and lack of adherence to medical provider recommendations. Eastern Thailand has the highest rate of human rabies deaths in the country [13,15]. Here we report on a descriptive epidemiological study of data from R36, for patients seeking care for a suspected rabies exposure from 1^st^ January to 31^st^ December 2015 in Eastern Thailand. This study aims to evaluate the representativeness of the R36 system, describe the characteristics of suspected rabies exposure among persons seeking medical care, describe utilization of rabies PEP and explore factors related to discontinuation of PEP against medical advice.

## Methods

**Fig 1:**
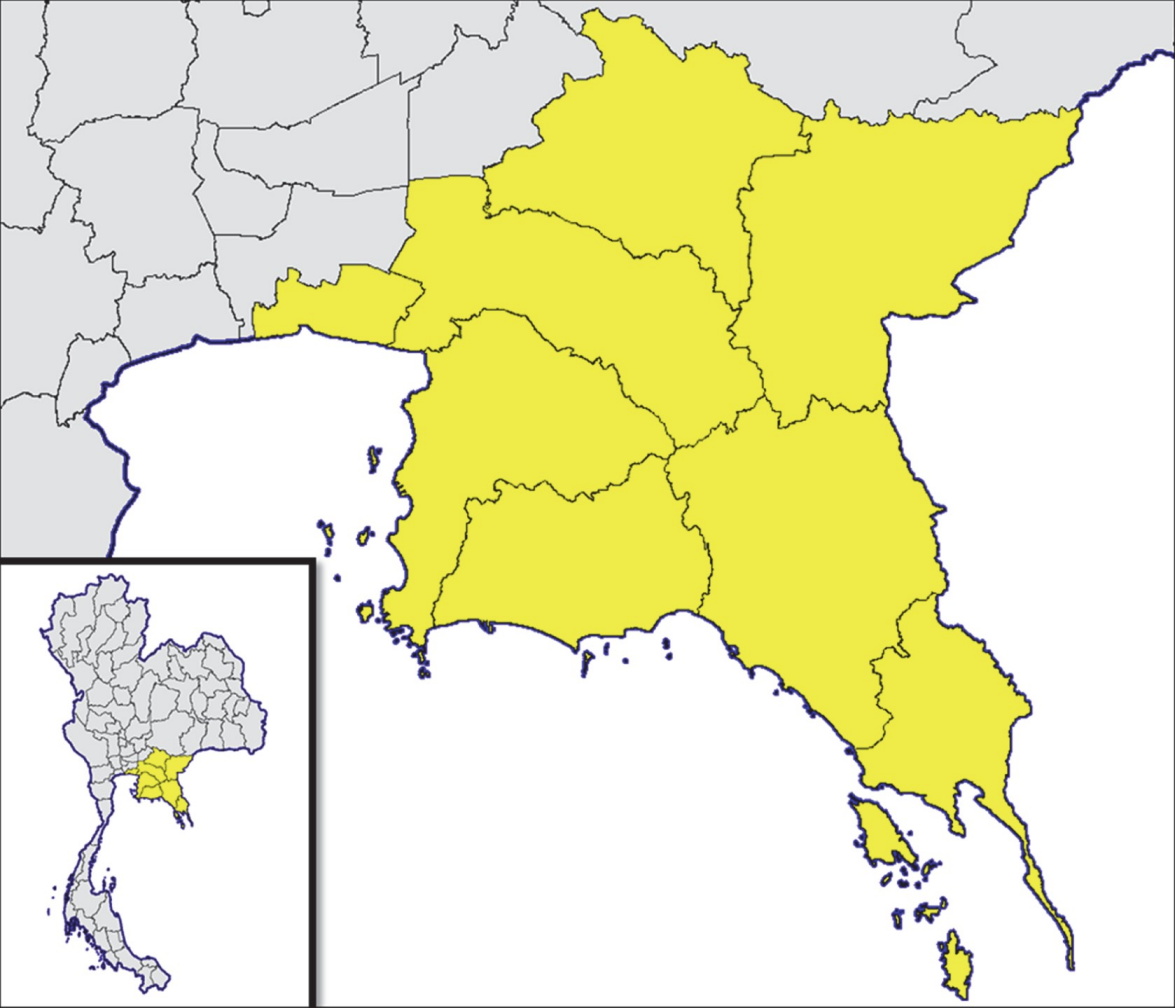
Map displaying the location of the eight provinces in Thailand evaluated for frequency and cause of suspected rabies exposures, 2015.

Data from the voluntary, electronic reporting platform R36 was analyzed in seven Eastern Thailand provinces (Chonburi, Rayong, Chanthaburi, Trat, Chachoengsao, Prachinburi and Sakaew) and one adjoining province in Central Thailand (Samut Prakarn) (Fig 1). The eight provinces included in this study have a cumulative human population of 5,840,308 (10% of Thailand population), and represent a geographical area of 35,394 kilometer square [16,18]. Reports from all persons seeking care for a suspected rabies exposure from January 1 –December 31, 2015 in hospitals which utilize R36 were analyzed.

The R36 platform collects demographic data of the exposed person, date of exposure, risk factors of the biting animal, severity of the bite, treatment recommended, and adherence to the PEP regimen (more information on the R36 system is available at http://r36.ddc.moph.go.th/r36/home). R36 is web-based, and can track patient data across any hospital which utilizes the platform; if persons seek care at multiple health centers their data is linked through a unique patient identification number. However, use of this database is voluntary, and currently it is used by 47.5% of hospitals in Eastern Thailand. All data analyzed were de-identified to maintain anonymity of those who sought medical care for a suspected rabies exposure.

Data were cleaned and analyzed in Epi-info 7.1.5.2. Descriptive analysis (ie frequencies) of demographic data, province, anatomical location of the exposure, predisposing cause of exposure (such as provoking behavior), animal type, animal owner status, animal age, animal vaccination history, post-bite therapies (ie washing) and PEP adherence were analyzed. Chi-square test was used to determine if the measure of association was statistically significant. A multivariable logistic regression model was developed in STATA/SE 11.0 to explore the associations between discontinuation of PEP and the aforementioned explanatory variables. Backwards selection based upon the log-likelihood ratio was applied to reach the most parsimonious model. Age-stratified rates of reported exposures were calculated based on human population census data and reported in rates per 100,000 people. Provincial population data was obtained from the Thailand-Official Statistic Registration System [16,18–19].

A multivariable linear regression model was developed to assess the degree of potential under-detection of suspected rabies exposures in Eastern Thailand. Provincial-level variables that were available for consideration in the regression analysis were: percentage of hospitals that utilized R36, province population density, number of hospitals per square kilometer, number of hospitals per 100,000 population, and average annual income per household. Average household income was used as a proxy for relative poverty differences between provinces. Backwards elimination was used to remove variables that did not significantly contribute to the regression model. Bivariate analysis indicated that Sa Kaew province had an unusually low rate of reported exposures in relation to hospital R36 participation. Therefore, two models were developed, one with and one without Sa Kaew province. Table 2 shows results from the model with the best fit based on r-square and root mean square error. Linear regression was conducted in SAS 9.2.

**Table 2:** Number of suspected rabies exposure that report to R36 and proportion of reported number by province.

Province	Human Population	Model Parameters				Number of Suspected Rabies Exposures			Rate of Rabies Exposures *(per 100*,*000 people)*		
		Proportion of Hospitals Utilizing R36	Population Density *(per km2)*	Hospitals *(per 100*,*000 people)*	Annual Household Income *(Baht)*	Reported through R36	Modeled	Modeled *(full participation)*	Reported through R36	Modeled	Modeled *(full participation)*
Sa Kaew*	544,849	89%	76	1.7	$ 26,953	40	702	766	7	129	141
Samut Prakan	1,255,175	27%	1,250	2.4	$ 25,457	500	470	1,442	40	37	115
Prachin Buri	475,365	20%	100	2.1	$ 24,166	573	331	734	121	70	154
Rayong	676,897	21%	191	2.1	$ 30,315	890	1,088	1,655	131	161	244
Chachoengsao	692,609	77%	129	2.5	$ 27,555	1,392	1,678	1,847	201	242	267
Chantaburi	524,330	54%	83	2.5	$ 36,204	1,967	1,891	2,147	375	361	409
Trat	216,083	88%	76	3.7	$ 25,333	842	876	904	390	406	418
Chon Buri	1,455,000	0%	333	2.0	$ 27,257	-	891	2,433	-	61	167
**Total**	**5,840,308**	**37%**	**165**	**2.2**	**$ 27,905**	**6,204**	**7,929**	**11,928**	**106**	**136**	**204**

* Sa Kaew Province was not included in model development

** FINAL MODEL: Provincial Exposure Rate = -648 + 106*(Proportion of Hospitals Utilizing R36) + -0.092*(Population Density) + 151.9*(Hospitals per 100,000) + 0.016*(Annual Household Income)

To estimate the degree of underreporting the final linear model parameter values were used to calculate provincial-level estimated exposure rates, with the model parameter “percentage of hospitals utilizing R36” set to 100%. The model output was interpreted as the adjusted rate of reported suspect rabies exposures had all hospitals utilized the R36 system. This number was then compared to the reported rate derived from entries into the R36 system.

The following surveillance system definitions were used in this analysis:

### A. Category of Rabies Exposure

A person who has had direct contact with a rabies suspect animal (any animal that is susceptible to rabies), as defined by the rabies Clinical Practice Guideline, Thailand Ministry of Health [12].

**Category I**: touching or feeding rabies suspect animals, licks on intact skin
**Category II**: nibbling of uncovered skin, minor scratches or abrasions without bleeding, lick on superficial broken skin
**Category III**: single or multiple transdermal bites or scratches, licks on deep broken skin from animals suspected to have rabies; contamination of mucous membrane with saliva from licks, contacts with bats

### B. Post-exposure prophylaxis

According to Thailand national policy all persons with category II and III exposures are recommended to initiate treatment immediately.

**Discontinued PEP Appropriately**:
A person with a suspected rabies exposure in category II or III for which the animal was shown not to have rabies through 10-day observation or laboratory testing, and PEP was discontinued.
**Discontinued PEP Inappropriately**:
A suspected rabies exposure in category II or III with no prior history of rabies vaccination who received less than four doses by IM or ID route
**Complete PEP**:
A person with a suspected rabies exposure in category II or III with no prior history of rabies vaccination who received at least four doses of rabies vaccine by either IM or ID route

### C. Exclusion criteria

R36 collects 23 variables; if more than 30% of variables were incomplete, the record was removed from analysis (Appendix 1). No records were excluded based on this criterion.

## Results

### Characteristic of animal exposure

**Fig 2:**
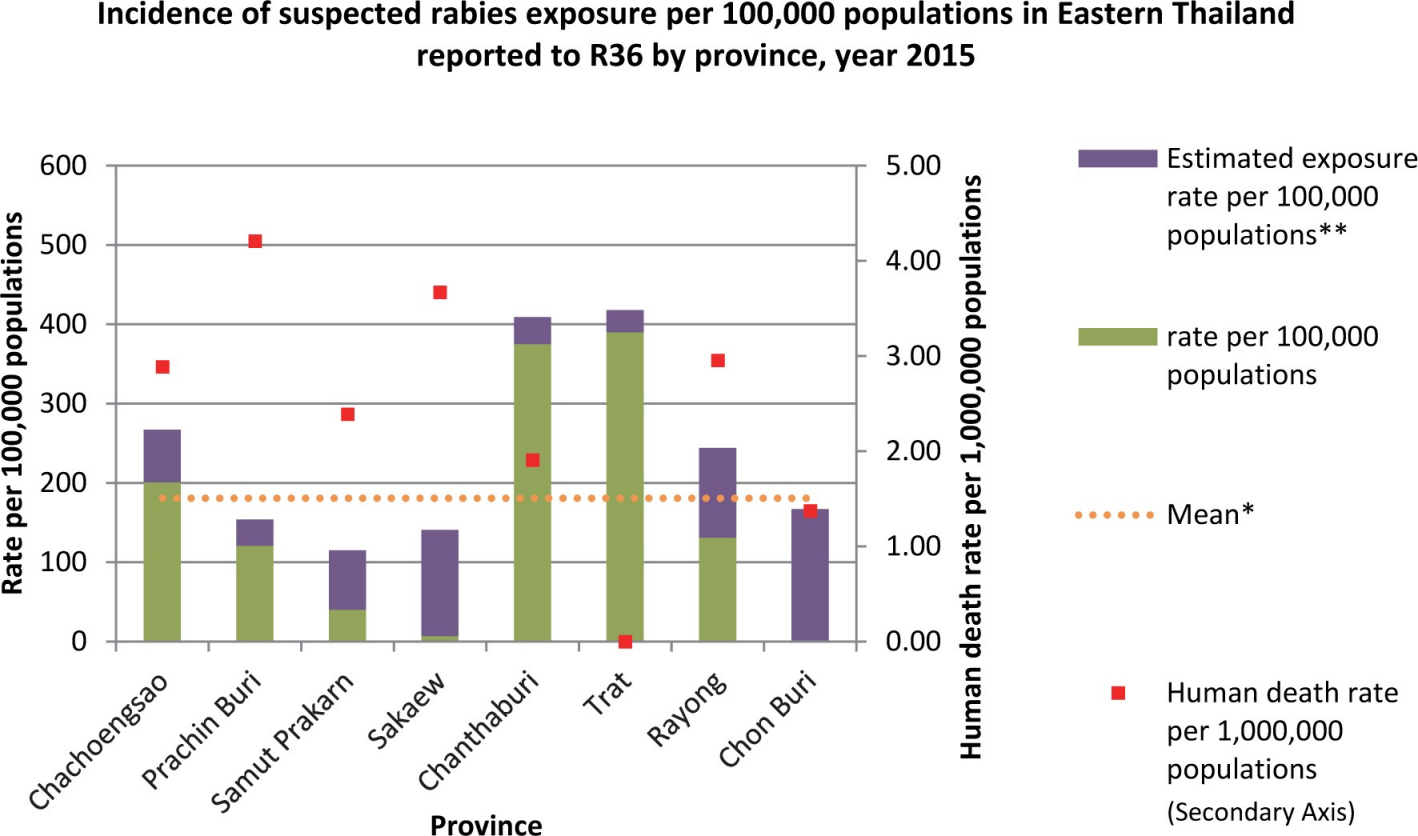
Incidence of suspected rabies exposure per 100,000 population in 8 Eastern Thailand provinces reported to R36 between 1^st^ Jan– 31^st^ Dec 2015. **Note**: * Crude mean; ** Estimation of suspected rabies exposure rate per 100,000 populations with full participation from predicted linear regression.

During 2015, R36 was operational in 48 of 130 (37%) hospitals in the study area. From 1^st^ January– 31^st^ December 2015, 6,204 suspected rabies exposures were reported from these 48 hospitals. The median reported exposure rate was 106 suspected exposures per 100,000 population (Province range: 0–390 exposure per 100,000 population per year). The provinces of Chantaburi and Trat had the highest reported rates of suspected rabies exposures (375 and 390 per 100,000 population, respectively) (Fig 2). No exposures were reported from Chonburi province.

The estimated rate of hospital-treated suspected rabies exposures was obtained through a linear regression model, which after backwards selection included the variables: proportion of hospitals utilizing R36, province population density, number of hospitals per 100,000 population, and provincial-level average annual household income (R^2^ = 0.94, df = 4, P = 0.12). Increased reports of suspect rabies exposures was associated with increases in hospital R36 participation, hospital density (per 100,000 people), and household income (coefficients 106, 152, and 0.016 respectively). Exposure reporting was negatively associated with human population density (coefficient -0.092). After extrapolation to an assumed 100% hospital R36 participation rate, the expected rate of hospital-based rabies exposures for the eight provinces was 204/100,000; a 1.9-fold increase over what was captured through R36 (Table 2).

The characteristics of suspected rabies exposures are shown in Table 3. Among total exposures, 91.8% were classified as category III. Legs (47.0%) and hands (21.2%) were the most common sites for category III exposures. Nearly 52% of persons reporting to hospitals for a suspected rabies exposure were female. Extremities of age had higher rates of reported exposures, with 1,651 persons (26.6%) aged <15 years (exposure rate 189.7 per 100,000 within age group) and 1,065 persons (17.2%) aged >60 years (exposure rate 189.2 per 100,000 within age group) (Table 4). The majority of suspected exposures were the result of unprovoked interactions with the animal (54.3%). Seventy percent of persons washed their wound before arriving at the hospital. Dogs were the most common source of exposure (77.8%) while 19.0% were cats. Seventy-seven percent of animals involved in a suspected rabies exposure were known to be owned. Only 22% of animals involved in a suspected rabies exposure had a history of rabies vaccination; vaccination status was unknown for the majority of animals (52.7%).

**Table 3:** Characteristic of suspected rabies exposures in Eastern Thailand reported to R36, 1^st^ Jan– 31^st^ Dec 2015.

Demographic and Risk Factors	No. of reported exposures (n = 6,204)	Percent	Category n, (%)		
			I n = 75 (1.2%)	II n = 434 (7.0%)	III n = 5,695 (91.8%)
Sex - Male - Female - N/A	2,781 3,209 214	44.8 51.7 3.5	39 (52.0) 36 (48.0) 0	181 (41.7) 231 (53.2) 22 (5.1)	2,561 (44.9) 2,942 (51.6) 192 (3.4)
Exposure site - Head/face/neck - Hand (s) - Arm (s) - Trunk - Leg (s) - Foot (s)	394 1,315 639 287 2,860 709	6.4 21.2 10.3 4.6 46.1 11.4	2 (2.7) 33 (44.0) 7 (9.3) 15 (20.0) 6 (8.0) 12 (16.0)	46 (10.6) 76 (17.5) 65 (14.9) 28 (6.5) 174 (40.1) 45 (10.4)	346 (6.1) 1,206 (21.2) 567 (9.9) 244 (4.3) 2,680 (47.0) 652 (11.4)
Predisposing cause - Provoked - Unprovoked - Unknown	2,727 3,366 111	44.0 54.3 1.8	18 (24.0) 53 (70.7) 4 (5.3)	185 (42.6) 241 (55.5) 8 (1.9)	2,524 (44.3) 3,072 (53.9) 99 (1.8)
Wound cleansing before arrived hospital					
- Yes - No - Unknown	4,220 1,967 17	68.1 31.7 0.8	19 (25.3) 55 (73.3) 1 (1.4)	298 (68.7) 134 (30.9) 2 (0.4)	3,903 (68.5) 1,778 (31.3) 8 (0.2)
Animal - Dog - Cat - Rat/Rabbit - Human - Other	4,824 1,181 97 11 91	77.8 19.0 1.6 0.2 1.5	65 (86.7) 4 (5.3) 0 (0.0) 5 (6.7) 1 (1.3)	294 (67.7) 130 (29.9) 3 (4.0) 0 (0.0) 7 (9.4)	4,465 (78.4) 1,047 (18.4) 94 (1.7) 6 (0.1) 83 (1.4)
Animal owner status					
- Owned - Not owned - Unknown	4,800 992 412	77.4 16.0 6.6	49 (65.3) 24 (32.0) 2 (2.7)	322 (74.2) 90 (20.7) 22 (5.1)	4,429 (77.8) 878 (15.4) 388 (6.8)
Animal vaccination history					
- At least once - Never - Unknown	1,384 1,552 3,268	22.3 25.2 52.7	12 (16.0) 25 (33.3) 38 (50.7)	79 (18.2) 127 (29.3) 228 (52.5)	1,293 (22.7) 1,400 (24.6) 3,002 (52.7)

**Table 4:** Suspected rabies exposures and dog bites reported through R36 surveillance system by age group, 1^st^ Jan– 31^st^ Dec 2015.

Age Group	Age population (Eastern Thailand)	All exposure categories		Dog bite exposures (Category III)	
		Number	Rate/100,000 populations	Number	Rate/100,000 populations
**0–15**	870,430	1,651	189.7	1,207	138.7
**16–30**	938,751	1,012	107.8	714	76.1
**31–45**	1,101,248	1,135	103.1	827	75.1
**46–60**	911,943	1,341	147.1	948	104.0
**>60**	562,936	1,065	189.2	769	136.6
Total**	4,385,308	6,204	141.5	4,465	101.8
Adjusted total*			204.2		
Median age = 37 years (0–96 years)					

Note

* Adjusted total was derived from linear regression model as described in the methods.

**Province of Chon Buri was not included in this analysis, as no hospitals participated in the R36 surveillance program

### Post-exposure prophylaxis (PEP) utilization

**Fig 3:**
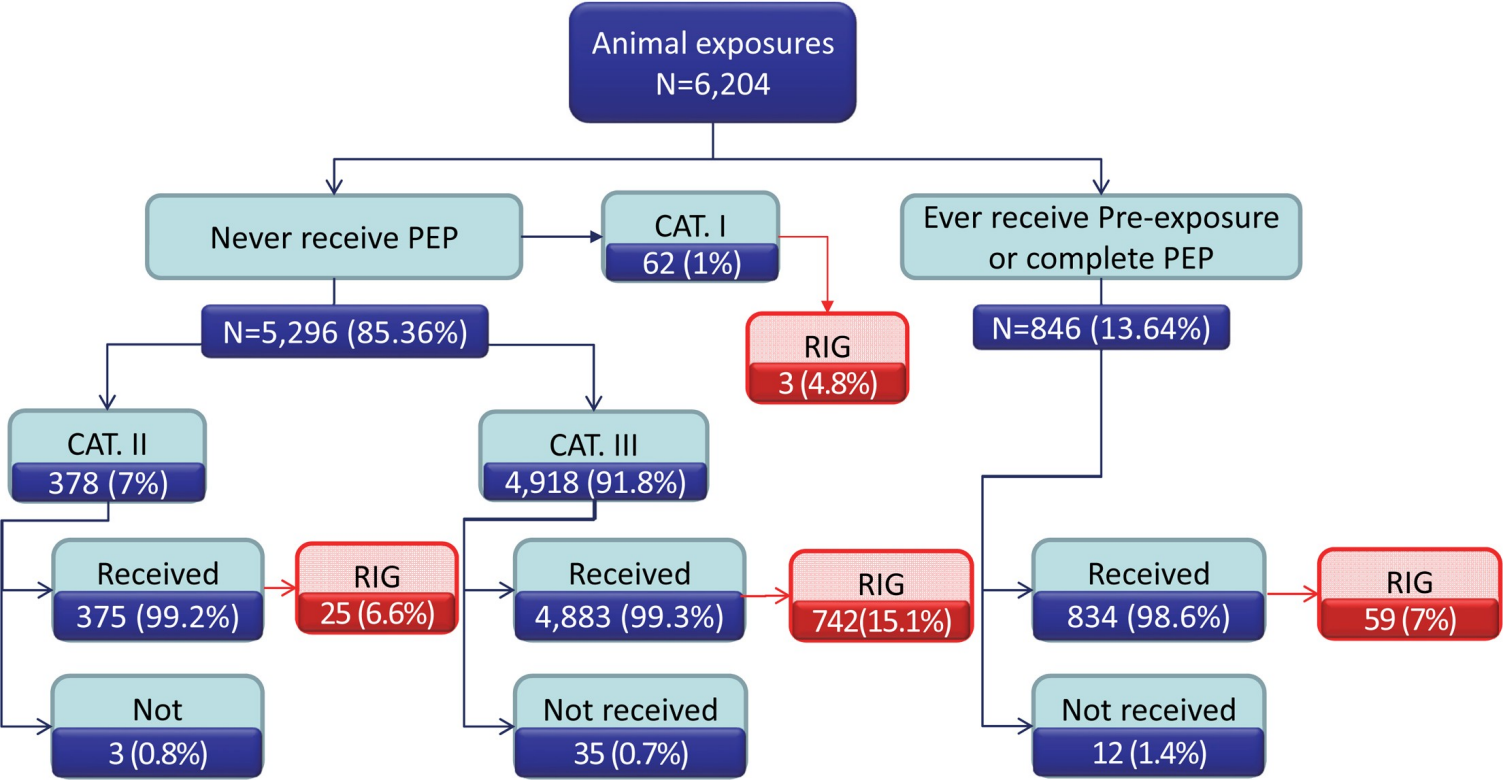
Distribution of rabies vaccine and immune globulin by rabies exposure category, as reported by R36 surveillance system, 1^st^ Jan– 31^st^ Dec 2015.

Among 6,204 exposures, 85.4% had never received previous rabies vaccination. Ninety-two percent and 7% were classified as category III and II exposures, respectively. Only 15% of persons with category III suspected rabies exposures received RIG. Approximately 1% of category II and III did not receive PEP (Fig 3).

## PEP completeness

Among the 5,296 persons with category II or III exposures who had no history of prior rabies vaccination, only 38 (1%) did not initiate PEP, contrary to medical advice (Fig 3). Adherence to rabies PEP is shown in Table 5. Of the 1,482 persons who initiated the 5-dose IM PEP regimen, 1,239 (83.6%) completed the second scheduled dose, 1,037 (70.0%) completed the third scheduled dose, 335 (22.6%) completed the fourth scheduled dose, and 234 (15.8%) completed the fifth scheduled dose. Of the 3,401 persons who initiated the 4-visit ID PEP regimen, 2,702 (79.4%) completed vaccination at the second visit, 2,352 (69.2%) completed vaccination at the third visit, and 1,581 (46.5%) completed vaccination at the fourth visit. Adherence through at least 4 doses (visits) was significantly better for persons who received the ID route (P-value <0.01). Of 846 persons with prior history of rabies vaccination, 12 did not initiate booster vaccination.

**Table 5:** Adherence to intramuscular and intradermal post-exposure prophylaxis among persons with category III exposures for whom 5-doses IM or 4-doses ID regimen of PEP was advised, 1^st^ Jan– 31^st^ Dec 2015.

Dose	Recommended Day of Vaccination Post-Bite	IM route (n = 1,482)					Day	ID route (n = 3,401)				Odds ratio	P-value***
		No. of received (%)	No. of delayed* (%)	Avg. Days Delayed**	Range (Days)	Dose		No. of received (%)	No. of delayed* (%)	Avg. Days Delayed**	Range (Days)		
1	0	1,482 (100)	181 (12.2)	0.4	0–160	1 & 2	0	3,401 (100)	311 (10.5)	0.8	0–396	0	0.99
2	3	1,239 (83.6)	53 (4.3)	3.1	3–12	3 & 4	3	2,702 (79.4)	87 (4.0)	3.2	3–30	0.95	0.99
3	7	1,037 (70.0)	37 (3.6)	4.1	4–29	5 & 6	7	2,352 (69.2)	42 (2.5)	7.1	7–31	0.99	0.99
4	14	335 (22.6)	14 (4.2)	7.3	7–23								
5	30	234 (15.8)	7 (3.0)	14.1	30–106	7 & 8	30	1,581 (46.5)	82 (4.8)	29.8	30–108	2.1	<0.01

Note

* No. of delayed = number of persons did not receive vaccine on the recommended date. For the day 0 dose, this refers to the number of days from the exposure until the first dose of vaccine was provided.

** First dose delay (in days) = bite date– 1^st^ dose date received

Second dose delay (in days) = 1^st^ dose date received– 2^nd^ dose date received

Third dose delay (in days) = 2^nd^ dose date received– 3^rd^ dose date received

Fourth dose delay (in days) = 3^rd^ dose date received– 4^th^ dose date received

*** The comparison of IM and ID adherence percentage by using Chi-square test

### Factors associated with discontinuing PEP

Factors associated with the discontinuation of PEP against medical advice are shown in Table 6. Males were more likely to discontinue PEP than females (p<0.001). Persons aged 16–30 and 30–45 were more likely to discontinue the PEP series compared to all other age categories (P<0.002). Exposures who did not receive RIG were more likely to discontinue PEP (p = 0.01). Victims bitten by animals other than dogs, such as monkeys or rodents, were less likely to discontinue compared to those bitten by dogs. People who were bitten by owned animals were more likely to discontinue PEP compared to those bitten by unowned animals (P<0.001). Person who were bitten due to provocation were more likely to discontinue PEP than persons who experienced an unprovoked bite (P<0.001). Those vaccinated by intramuscular route were prone to discontinue PEP compared to intradermal route (P<0.001). There was a large degree of variation in probability of discontinuing the vaccination series by province, with discontinuation risks highest in Prachiburi.

**Table 6:** Factors associated with PEP completeness in category III exposure that required 4 (intradermal) or 5 (intramuscular) doses of PEP, 1^st^ Jan– 31^st^ Dec 2015.

Factor	Discontinued Series Against Medical Advice (%)	Completed Vaccination Series (%)	Adjusted OR (C.I.)	P-value
Sex - Female (n = 2,494) - Male (n = 2,210) - Unreported (n = 179)	1,556 (62.4) 1,453 (65.7) 59 (33.0)	938 (37.6) 757 (34.3) 120 (67.0)	Referent 1.3 (1.1–1.5) 0.7 (0.5–1.0)	Referent 0.008 0.03
Age group - 0–15 years (n = 1,309) - 16–30 years (n = 823) - 30–45 years (n = 921) - 45–60 years (n = 1,032) - > 60 years (n = 798)	775 (59.2) 586 (71.2) 611 (66.3) 619 (60.0) 477 (59.8)	534 (40.8) 237 (28.8) 310 (33.7) 413 (40.0) 321 (40.2)	Referent 1.8 (1.4–2.2) 1.4 (1.1–1.7) 1.1 (0.9–1.3) 0.9 (0.7–1.1)	Referent <0.0001 0.002 0.44 0.38
Province - Samut Prakarn (n = 378) - Rayong (n = 747) - Chanthaburi (n = 1,675) - Trat (n = 673) - Chachoengsao (n = 990) - Prachinburi (n = 386) - Sakaew (n = 34)	199 (52.6) 299 (40.0) 1,418 (84.7) 463 (68.8) 337 (34.0) 349 (90.4) 3 (8.8)	179 (47.4) 448 (60.0) 257 (15.3) 210 (31.2) 653 (66.0) 37 (9.6) 31 (9.2)	0.2 (0.1–0.2) 0.3 (0.3–0.4) 1.6 (1.2–2.0) Referent 0.2 (0.2–0.3) 6.3 (4.2–9.5) 0.0 (0.0–0.1)	<0.0001 <0.0001 0.0001 Referent <0.0001 <0.0001 <0.0001
RIG - Received (n = 730) - Not received (n = 4,153)	596 (94.1) 2,472 (59.5)	134 (18.4) 1,681 (40.5)	0.7 (0.5–0.9) Referent	0.01 Referent
Exposure site* - Head/face/neck (n = 304) - Extremities (n = 4,366) - Trunk (n = 213)	213 (70.1) 2,736 (62.7) 119 (55.9)	91 (29.9) 1,630 (37.3) 94 (44.1)	-	-
Animal age - Unknown (n = 2,067) - < 3 month (n = 127) - 3–12 month (n = 537) - >12 month (n = 2,152)	1,530 (74.2) 68 (53.5) 259 (48.2) 1,211 (56.3)	537 (25.8) 59 (46.5) 278 (51.8) 941 (43.7)	-	-
Animal type - Dog (n = 3,857) - Cat (n = 873) - Other (n = 153)	2,443 (63.3) 542 (62.1) 83 (54.2)	1,414 (36.7) 331 (37.9) 70 (45.8)	Referent 1.0 (0.8–1.2) 0.4 (0.3–0.6)	Referent 0.99 <0.0001
Owner status - Not owned (n = 769) - Owned (n = 3,776) - Unknown (n = 338)	429 (55.8) 2,386 (63.2) 253 (74.9)	340 (44.2) 1,390 (36.8) 85 (25.1)	Referent 1.6 (1.3–1.9) 1.0 (0.7–1.4)	Referent <0.0001 0.89
Provocation - Provoked (n = 2,218) - Not Provoked (n = 2,574) - Unknown (n = 91)	1,591 (71.7) 1,425 (55.34) 52 (57.1)	627 (28.3) 1,149 (44.6) 39 (42.9)	1.6 (1.3–1.9) Referent 1.2 (0.7–2.0)	<0.0001 Referent 0.41
Route - Intradermal (n = 3,401) - Intramuscular (n = 1,482)	1,820 (53.5) 1,248 (84.2)	1,581 (46.5) 234 (15.8)	Referent 4.8 (3.9–5.9)	Referent <0.0001

## Discussion

This study describes the rates of healthcare seeking behavior to hospitals for treatment of suspected rabies exposures through data collected by an electronic, voluntary, hospital-based reporting system: R36. Between 1^st^ January– 31^st^ December 2015, 6,204 suspected rabies exposures were reported through R36 in eight Eastern Thailand provinces. The percentages of hospitals utilizing R36 during this time-period was highly variable, 0% to 90% per province, indicating significant potential for under-detection of persons seeking medical care for rabies exposures. Furthermore, these are reported exposures, and it is not possible to determine how accurately this data reflects the true number of rabies exposures that occur in these eight provinces, which is assuredly much higher due to lack of healthcare seeking after exposure [20]. Despite these limitations, results from this study can help to improve policies that impact PEP access for persons with rabies exposures, as well as develop rabies prevention policies in Southeast Asia.

The very young and the very old were over-represented among the population of rabies-exposed persons reported in R36, as compared to the population as a whole [21–22]. This is consistent with what has been reported for rabies exposures in numerous other studies [23]. The increased frequency of reported exposures in these two extremes of the age categories can be explained by their behavioral interactions with dogs. Dogs tend to act in a dominant manner towards children because of their small size and children may not recognize when a dog is sick (ie rabid) or when a dog is prone to bite [24]. Furthermore, children lack the ability to fend off attacks by animals and sometimes bites may take place with provocation from children through antagonistic interactions (i.e. stone throwing, beating, chasing or running at the sight of the dogs)[25–26]. In Thailand the elderly are often the primary caretakers of family dogs, and they have good healthcare seeking behavior which could lead them to seek medical care at a higher rate than other age categories [27]. Similarly, parents of children bitten by animals may prioritize their healthcare, and seek medical care at higher rates compared to young adults. These findings re-inforce the global understanding that children’s interactions with dogs put them in the highest risk category for rabies exposure through bites.

Most of suspected rabies exposures were defined as Category III and the main source of exposure was dogs. Category III exposures may be overrepresented in hospital-based surveillance systems like R36 because they often detect serious injuries which required medical treatment, whereas category I and II exposures are minor injuries that are likely treated at home or in local clinics [23,28].

Only 15% of persons with Category III exposures received RIG, despite national recommendations for its provision to all Category III bite victims [29]. Thailand does produce both equine RIG (ERIG) and human RIG (HRIG), however domestic production is insufficient to meet demand. The Ministry of Health routinely imports HRIG and ERIG to address shortages, but this does not meet the demand when considering that RIG is recommended for all Category IIII exposures [30]. Despite domestic production and importation, supply limitations of ERIG and HRIG are often reported and may have led to reduced access for the patients represented in this study [30]. Another explanation for reduced RIG use among bite victims is that RIG costs 2,500 Thai baht per vial, the equivalent of one-week’s salary for an average Thai resident, and a cost that must be paid by the patient [31]. This cost may have led to a lack of adherence to medical recommendations. Thailand’s three-tiered healthcare system provides different levels of coverage for rabies PEP expenses, but unfortunately the R36 surveillance system does not collect information on the patient’s economic or insurance status and it does not collect information as to the cause of PEP discontinuation. Future studies should further explore the reasons for lack of PEP adherence, with attention to issues related to insurance coverage, costs to the patient, and perceived risk. Recent WHO guidance recommends that countries consider using monoclonal antibody cocktails to fill critical gaps in RIG availability. Monoclonal antibodies should be further evaluated as a potential solution for the apparent lack of availability in RIG [32].

Most persons with suspected rabies exposures discontinued PEP against medical advice, for both the ID and IM regimens, a finding that has been reported in other studies as well [33–34]. A substantial proportion of people received only one dose of vaccine (30%); future studies should explore factors that lead to unadvised discontinuation of PEP. While adherence to the PEP schedule was low, it was significantly better for persons who received vaccination from the ID route, as has been noted in other studies [29]. There were no explanations for adherence behavior between IM and ID route identified in this analysis, but this difference in PEP completion remained in the adjusted model, suggesting that the disparity is real and not due to confounding factors assessed in this study. Bite victims pay an average of 1,500–2,000 Thai baht for PEP [20], regardless of IM or ID administration, so cost is unlikely to be responsible for improved ID vaccination adherence. Abbreviated PEP schedules have been proposed to improve adherence to the PEP regimen; however in this study the four ID doses given over 30 days had better patient adherence than the fourth IM dose which is given on day 14. Anecdotally, the IM injection may be more painful than a properly placed ID injection, which may account for some improvements in adherence. Furthermore, associations between the type of facilities providing ID injection and adherence should be investigated, as it is possible that the catchment population or medical providers in these facilities are more knowledgeable about rabies prevention.

Discontinuing the PEP series, in the adjusted model, was associated with variables often attributed to low-risk exposures. Bite victims were more likely to discontinue if they were bitten by an owned dog, if it was a provoked bite, and if they did not receive RIG (typically prescribed for severe and high-risk bite events). This finding may be showing some degree of self-risk assessment, where bite victims do not complete the series when they believe the risk is low. It is also possible that additional factors not captured by R36 are informing these decisions, such as the discontinuation after passing of quarantine periods. Health officials should consider providing more structured risk assessment, animal investigation, and PEP discontinuation guidance. As less than half of bite victims appear to discontinue against medical advice, yet this has not resulted in any cases of human rabies to-date, there appears to be both a demand and justification to consider medical-provider driven risk-based approaches to PEP administration and discontinuation. The R36 system should consider collecting information when discontinuation of the PEP series is advised by a medical provider.

Previous publications have shown that fear of acquiring rabies and knowledge of the gravity of the disease, as well as the cost of vaccine were factors affecting PEP compliance [35]. In this surveillance analysis, persons exposed to owned dogs were much more likely to discontinue vaccination against medical advice. Persons with exposures to owned, who often bite as part of normal play behavior, may have trivialized these exposures due to a lack of fear or knowledge that pet can have rabies, which could lead to reduced PEP adherence. From the national animals rabies surveillance reporting system (Thai rabies net), around 50% of rabid dogs were owned [36]. Improved dissemination of these surveillance results and education on the risk of rabies in biting dogs may help improve knowledge of rabies and appropriate levels of fear regarding potential exposures, and thereby improve adherence to recommended vaccination schedules.

For conclusion, the voluntary, web-based R36 system can provide valuable feedback about patient adherence to medical advice and medical provider treatment recommendations. Fewer than 50% of hospitals utilized R36 during this study period, which may result in biased results during analysis. Given the patient tracking-capacity of the platform and utility for monitoring trends in rabies exposures and PEP adherence, more hospitals should consider utilizing R36. Moreover, to improve adherence to PEP, rabies exposure treatment training should be provided to healthcare workers. Education on rabies vaccination should be provided to bite victims, particularly persons in high risk groups such as children, and persons who provide care to owned and community dogs. Hospitals utilizing R36 should consider active outreach to patients that discontinue PEP against medical advice, to encourage their return for vaccination. Regarding to the PEP regimen, RIG is not being properly utilized. Further evaluations should be made in regard to the procurement, distribution, and disbursement of RIG in Thailand. The national program should consider human RIG alternatives such as potentially laboratory-derived rabies immune-globulin products, as well as programs to make these products affordable to persons with qualifying exposures. The ID route has shown superior adherence when compare to the IM route. Further investigation into the factors related to improved adherence should be evaluated, including cost of the PEP series, pain associated with IM administration, or other unspecified factors.

However, there are limitations according to this study as R36 is not a mandatory reporting system, and only 42% of hospitals in Eastern Thailand participated as of 2015. Furthermore, the system may not capture persons who transfer from an R36 to a non-R36 hospital during the course of their treatment. If there are non-random factors that led to hospital participation and patient tracking then the results reported here may be biased. However, the data collected by R36 represents some of the most detailed PEP and rabies exposure data that is systematically collected, and the findings can be used to improve rabies control in Thailand and other countries that may have similar healthcare systems.

## Supporting information

S1 Data Extraction Form (DOCX)
