# Supplementary material for: An epidemiological study of suspected rabies exposures and adherence to rabies post-exposure prophylaxis in Eastern Thailand, 2015
Source: PLoS Negl Trop Dis. 2020 Feb 27;14(2):e0007248. doi: 10.1371/journal.pntd.0007248 (PMC7077841; doi:10.1371/journal.pntd.0007248)
Supplement: S1 Data Extraction Form — (DOCX) [file pntd.0007248.s001.docx]

**S1**

**Data extraction form**

| Record number | ***** |
| --- | --- |
| Year | **** |
| Hospital number | ********** |
| Citizen ID | ************* |
| Province code | ** |
| Age | ** |
| Sex | * (1 = Male, 2 = Female) |
| Nationality | * (1 = Thai, 2 = Other, 9 = Unknown) |
| Bite date | dd/mm/yyyy |
| Bite site | * (1 = head/neck, 2 = hand, 3 = Arm(s), 4 = trunk, 5 = leg(s), 6 = foot(s) |
| Category | * |
| Bleed | * (1 = Yes, 2 = No) |
| Animal type | * (1 = Dog, 2 = Cat, 3 = Rodent, 4 = Other, 5 = Human) |
| Animal age | * (1 = <3 month, 2 = 3-6 month, 3 = 6-12 month, 4 = >12 month, 5 = Unknown) |
| Owner | * (1 = Owned, 2 = Not owned, 3 = Unknown) |
| Animal rabies vaccination history | * (1 = Unknown, 2 = Never received vaccination, 3 = Ever received once in lifetime, 4 = Ever receive within 1 year, 5 = Ever received once more than 1 year) |
| Bite cause | * (1 = With cause such as provoke, 2 = Without cause) |
| Wound cleansing | * (1 = Yes, 2 = No) |
| Antiseptic application | * (1 = Yes, 2 = No) |
| Human rabies vaccination history | * (1 = Never received PEP or Prep, 2 = Ever received PEP or Prep within 6 month, 3 = Ever received PEP or Prep more than 6 month) |
| Immunoglobulin | * (1 = Yes, 2 = No) |
| Vaccination route | * (1 = IM, 2 = ID, 3 = Not received) |
| First dose date | dd/mm/yyyy |
| Second dose date | dd/mm/yyyy |
| Third dose date | dd/mm/yyyy |
| Forth dose date | dd/mm/yyyy |
| Fifth dose date | dd/mm/yyyy |
